# Cross-sectional analysis of cardiovascular disease and risk factors in patients with spondyloarthritis: a real-life evidence from biostar nationwide registry

**DOI:** 10.1007/s00296-023-05523-y

**Published:** 2024-02-06

**Authors:** Mehmet Tuncay Duruöz, Hatice Bodur, Şebnem Ataman, Gülcan Gürer, Özgür Akgül, Hasan Fatih Çay, Erhan Çapkın, İlhan Sezer, Aylin Rezvani, Meltem Alkan Melikoğlu, İlker Yağcı, Fatma Gül Yurdakul, Feride Nur Göğüş, Ayhan Kamanlı, Remzi Çevik, Lale Altan

**Affiliations:** 1 Division of Rheumatology, Department of Physical Medicine and Rehabilitation, Marmara University School of Medicine, Istanbul, Türkiye; 2 Department of Physical Medicine and Rehabilitation, Faculty of Medicine, Yıldırım Beyazıt University, Ankara City Hospital, Ankara, Türkiye; 3 Division of Rheumatology, Department of Physical Medicine and Rehabilitation, Ankara University School of Medicine, Ankara, Türkiye; 4 Division of Rheumatology, Department of Physical Medicine and Rehabilitation, University School of Medicine, Adnan Menderes University, Aydın, Türkiye; 5 Division of Rheumatology, Department of Physical Medicine and Rehabilitation, Manisa Celal Bayar University School of Medicine, Manisa, Türkiye; 6 Division of Rheumatology, Department of Physical Medicine and Rehabilitation, Antalya Training and Research Hospital, Antalya, Türkiye; 7 Department of Physical Medicine and Rehabilitation, Karadeniz Technical University School of Medicine, Trabzon, Türkiye; 8 Division of Rheumatology, Department of Physical Medicine and Rehabilitation, University School of Medicine, Akdeniz University, Antalya, Türkiye; 9 Department of Physical Medicine Rehabilitation, Internatonal School of Medicine, İstanbul Medipol University, Istanbul, Türkiye; 10 Department of Physical Medicine Rehabilitation, School of Medicine, Atatürk University, Erzurum, Türkiye; 11 Department of Physical Medicine Rehabilitation, University School of Medicine, Marmara University, Istanbul, Türkiye; 12 Department of Physical Medicine and Rehabilitation, Ankara City Hospital, University of Health Sciences, Ankara, Türkiye; 13 Department of Physical Medicine Rehabilitation and Rheumatology, Gazi University School of Medicine, Ankara, Türkiye; 14 Department of Physical Medicine Rehabilitation and Rheumatology, School of Medicine, Sakarya University, Sakarya, Türkiye; 15 Department of Physical Medicine Rehabilitation, School of Medicine, Dicle University, Diyarbakır, Türkiye; 16 Department of Physical Medicine Rehabilitation and Rheumatology, School of Medicine, Uludağ University, Bursa, Türkiye

**Keywords:** Spondyloarthritis, Axial, Psoriatic arthritis, Cardiovascular risk, Prevalence

## Abstract

The association between spondyloarthritis and cardiovascular (CV) diseases is complex with variable outcomes. This study aimed to assess the prevalence rates of CV diseases and to analyze the impact of CV risk factors on CV disease in patients with spondyloarthritis. A multi-center cross-sectional study using the BioSTAR (Biological and Targeted Synthetic Disease-Modifying Antirheumatic Drugs Registry) database was performed on patients with spondyloarthritis. Socio-demographic, laboratory, and clinical data were collected. Patients with and without major adverse cardiovascular events (MACE) were grouped as Group 1 and Group 2. The primary outcome was the overall group’s prevalence rates of CV disease and CV risk factors. The secondary outcome was the difference in socio-demographic and clinical characteristics between the groups and predictive risk factors for CV disease. There were 1457 patients with a mean age of 45.7 ± 10.9 years. The prevalence rate for CV disease was 3% (*n* = 44). The distribution of these diseases was coronary artery disease (*n* = 42), congestive heart failure (*n* = 4), peripheral vascular disorders (*n* = 6), and cerebrovascular events (*n* = 4). Patients in Group 1 were significantly male (*p* = 0.014) and older than those in Group 2 (*p* < 0.001). There were significantly more patients with hypertension, diabetes mellitus, chronic renal failure, dyslipidemia, and malignancy in Group 1 than in Group 2 (*p* < 0.05). Smoking (36.7%), obesity (24.4%), and hypertension (13.8%) were the most prevalent traditional CV risk factors. Hypertension (HR = 3.147, 95% CI 1.461–6.778, *p* = 0.003), dyslipidemia (HR = 3.476, 95% CI 1.631–7.406, *p* = 0.001), and cancer history (HR = 5.852, 95% CI 1.189–28.810, *p* = 0.030) were the independent predictors for CV disease. A multi-center cross-sectional study using the BioSTAR (Biological and Targeted Synthetic Disease-Modifying Antirheumatic Drugs Registry) database was performed on patients with spondyloarthritis. Socio-demographic, laboratory, and clinical data were collected. Patients with and without major adverse cardiovascular events (MACE) were grouped as Group 1 and Group 2. The primary outcome was the overall group’s prevalence rates of CV disease and CV risk factors. The secondary outcome was the difference in socio-demographic and clinical characteristics between the groups and predictive risk factors for CV disease. There were 1457 patients with a mean age of 45.7 ± 10.9 years. The prevalence rate for CV disease was 3% (*n* = 44). The distribution of these diseases was coronary artery disease (*n* = 42), congestive heart failure (*n* = 4), peripheral vascular disorders (*n* = 6), and cerebrovascular events (*n* = 4). Patients in Group 1 were significantly male (*p* = 0.014) and older than those in Group 2 (*p* < 0.001). There were significantly more patients with hypertension, diabetes mellitus, chronic renal failure, dyslipidemia, and malignancy in Group 1 than in Group 2 (*p* < 0.05). Smoking (36.7%), obesity (24.4%), and hypertension (13.8%) were the most prevalent traditional CV risk factors. Hypertension (HR = 3.147, 95% CI 1.461–6.778, *p* = 0.003), dyslipidemia (HR = 3.476, 95% CI 1.631–7.406, *p* = 0.001), and cancer history (HR = 5.852, 95% CI 1.189–28.810, *p* = 0.030) were the independent predictors for CV disease. The prevalence rate of CV disease was 3.0% in patients with spondyloarthritis. Hypertension, dyslipidemia, and cancer history were the independent CV risk factors for CV disease in patients with spondyloarthritis.

## Introduction

Spondyloarthritis (SpA), recognized as a chronic inflammatory condition, encompasses various phenotypes, including ankylosing spondylitis [currently known as radiographic axial SpA (r-axSpA)], non-radiographic axial SpA (nr-axSpA), peripheral SpA, psoriatic arthritis, enteropathic SpA, reactive arthritis, and undifferentiated SpA [1–4]. While distinct disease characteristics define each SpA phenotype, typical clinical features and comorbidities are frequently observed in SpA patients, prompting the consideration of these diverse conditions as singular disease entities [3].

The association of SpA with cardiovascular (CV) risk factors and major adverse CV events (MACE), such as ischemic heart diseases and cerebrovascular events, has gained popularity for the last decades with conflicting findings [1, 2, 5–7]. In SpA patients, a 20–40% increase in CV mortality has been reported compared to the general population [8–10]. While certain CV risk factors like age, sex, and family history are non-modifiable, the five primary traditional and modifiable risk factors—hypertension, smoking, dyslipidemia, diabetes, and obesity—account for over 50% of all CV deaths in SpA patients [1, 2, 11, 12]. Therefore, identifying these factors and implementing control measures are crucial in managing the disease and averting morbidity and mortality in SpA patients [1, 13, 14].

Atherosclerosis-related CV events, such as ischemic heart disease, stroke, and myocardial infarction, are the prevalent disease conditions for SpA patients [5, 13–16]. Studies have indicated a positive correlation between the number of risk factors and the occurrence of CV disease (CVD) [2, 11, 17]. The complex relationship between CV risk factors, treatment approaches, disease-related features, and increased mortality in SpA patients warrants further exploration [1, 5, 11, 13]. Besides the considerable variations in the significance of such relationships for each country, the studies investigating these issues in the same patient groups, including all phenotypes of SpA, are limited in number [1, 9].

The inception of the BioSTAR (Biological and Targeted Synthetic Disease-Modifying Antirheumatic Drugs Registry) database by the Turkish League Against Rheumatism (TLAR) marks a groundbreaking initiative in Turkey, notably aimed at aggregating comprehensive real-life data about chronic conditions like SpA [18]. This nationwide registry stands as a pioneering effort, uniquely positioned to capture the intricate nuances of SpA's trajectory within the Turkish population, offering invaluable insights into its prevalence and prognostic determinants [19, 20]. Moreover, the intrinsic value of long-standing registries transcends mere data collection; they serve as indispensable tools for unraveling a disease's complex trajectory, assessing treatment outcomes, and discerning predictive factors for adverse events, efficacy, and safety of targeted therapeutic strategies [21]. Comprehensive analysis of registry-based cohorts, utilizing the extensive repository of real-world data, aims to uncover and elucidate the precise risk factors linked to CVD in patients with SpA.

This study aimed to assess the prevalence rate of CVD, analyze the demographic and clinical characteristics of SpA patients with and without documented MACEs, and investigate the impact of CV risk factors on the development of CVD in patients with all SpA phenotypes.

## Materials and methods

### Study

A multi-center cross-sectional study with follow-up data was conducted to investigate the prevalence rates of CVD and CV risk factors in patients with a diagnosis of SpA utilizing the database of the BioSTAR registry [18]. The local ethical committee approved the study (Turkey Medicines and Medical Devices Agency, 66,175,679–514.99–E.6366, and Ankara Numune Training and Research Hospital Ethics Committee, E-182413). Experienced physicians followed up with all patients at 6-month intervals and uploaded the updated clinical information to a predetermined electronic worksheet. All data in this database were evaluated in September 2022 for the current study). The study adhered to the Helsinki Declaration of 1964 principles and its subsequent amendments. All participants gave written informed consent before participating in the BioSTAR–SpA database.

### Patients

We included all adult patients (≥ 18 years) with a diagnosis of SpA according to the Assessment of SpondyloArthritis International Society (ASAS) classification criteria [22]. Patients with missing socio-demographic or clinical data were excluded. Comprehensive medical history, physical examination, and laboratory tests were conducted for all patients.

### Study variables

Socio-demographic data encompassed age, sex, body mass index (BMI), educational and marital status, smoking and alcohol status, comorbidities, and geographical regions of Turkey where the patients lived. The patients' BMI values were calculated as weight in kilograms divided by the square of height in meters (kg/m^2^). We categorized the patients with age (< 40 and ≥ 40 years) and BMI (≥ 30 kg/m^2^ and < 30 kg/m^2^). Laboratory investigations at the patients' last admission to attending centers were recorded.

The disease-related characteristics of the patients included the disease's duration, the SpA's phenotypes, and HLA B-27 status. The patients were grouped according to the phenotypes of SpA as r-axSpA, nr-axSpA, peripheral SpA, psoriatic arthritis, enteropathic SpA, reactive arthritis, and undifferentiated SpA [1]. The last measured results of the laboratory parameters, including C-reactive protein (CRP) and erythrocyte sedimentation rate (ESR), were recorded. The severity of the diseases was evaluated based on the scores of the Bath Ankylosing Spondylitis Disease Activity Index (BASDAI; 1–10) and Ankylosing Spondylitis Disease Activity Score with CRP (ASDAS–CRP). The BASDAI consists of six 10 cm horizontal visual analog scales (VAS) to measure fatigue severity, spinal and peripheral joint pain, localized tenderness, and morning stiffness. The final score has a range of 0–10 [23]. The scores of ≥ 4.0 for BASDAI and ≥ 2.1 for ASDAS–CRP were regarded as high-disease activity [12, 18]. The disease activity indexes and the patient-reported outcomes at the time of the last visit, including ASDAS–ESR, Maastricht Enthesopathy Score (MASES; 1–10), the Bath Ankylosing Spondylitis Functional Index (BASFI; 1–10), the global VAS (1–100) for the patient, the physician, pain, and fatigue, the Disease Activity Index for Psoriatic Arthritis (DAPSA), the Psoriasis Area and Severity Score (PASI), the symptom severity score, fibromyalgia severity score, and tender, and swollen joint counts, were recorded [18, 20, 23–29].

The MASES score is calculated considering tenderness on 13 enthesis sites, graded from zero (no pain) to three (wince or withdraw). The range is between zero and 39 [18]. A self-assessment instrument with eight questions was used to calculate the BASFI score. The answer to each question corresponded to a 10 cm horizontal VAS with a mean score range from zero to ten [24]. The DAPSA is a composite score calculated with the number of painful and swollen joints, the global VAS for patients (1–10) and pain (1–10), and CRP. Higher scores indicate higher disease activity for psoriatic arthritis [25].

The PASI is a clinician-rated score for psoriasis severity in four anatomic locations (head, upper limbs, trunk, and lower limbs). Depending on the disease severity and surface area involvement, the adjusted total scores ranging from zero to 72 were calculated for each patient. Higher scores indicate more severe psoriatic conditions [26, 27].

The symptom severity score was determined considering fatigue, unrefreshing sleep, cognitive manifestations, and somatic symptoms, assigning from zero (no symptoms) to three (a great deal of symptoms) based on severity or amount. The total score was 12 [28].

The fibromyalgia severity score is calculated by summating the widespread pain index and the symptom severity scores. The range is between zero (no symptoms) and 31 (most severe symptoms). A higher score is regarded as an approximate measure of fibromyalgia severity [29].

The medication use and switching status were searched using the patient's medical files.

### Traditional CV risk factors

The medical records of the patients were explicitly searched to ascertain the presence of CV risk factors: (1) dyslipidemia (physician diagnosis, or the use of lipid-lowering medication, or at least one factor: total cholesterol > 200 mg/dL, triglycerides > 150 mg/dL, HDL-cholesterol < 40 mg/dL in men or < 50 mg/dL in women, or LDL-cholesterol > 130 mg/dL), (2) hypertension (physician diagnosis and/or use of anti-hypertensive medications), (3) obesity (BMI ≥ 30 kg/m^2^), (4) currently active smoking, and (5) diabetes mellitus (physician diagnosis, or glycemia > 126 mg/dL, HbA1c > 6.5%, or glucose-lowering drugs or insulin therapy) [2, 30–32].

### Groups

The patients were grouped according to the MACE category's proven diagnoses of CV events (Group 1). We used a composite definition of MACE for myocardial infarction, ischemic heart disease, peripheral vascular disorders, congestive heart failure, ischemic stroke, and transient ischemic attack [33, 34]. All these diseases were included in the definition of CVD. Group 2 included patients without MACE.

### Statistical analysis

The primary outcome was the prevalence rates of CVD and CV risk factors in patients with SpA. The secondary outcomes were the differences in socio-demographic and clinical characteristics between patients with and without CVD and the identification of the potential risk factors in predicting the development of CVD in the study group.

For descriptive statistics, mean ± standard deviation was used to present continuous data with normal distribution. Median with minimum–maximum values was applied for continuous variables without normal distribution. Numbers and percentages were used for categorical variables. The Shapiro–Wilk and Kolmogorov–Smirnov tests analyzed the normal distribution of the numerical variables.

The ındependent samples *t* test compared two independent groups where numerical variables had a normal distribution. The Mann–Whitney *U* test was applied for the variables without normal distribution in comparing two independent groups. The Pearson Chi-square and Fisher's exact tests were used to compare the differences between categorical variables in 2 × 2 tables.

The univariable and multivariable Cox proportional hazard regression models were used to estimate the crude hazard ratios (HRs) and 95% confidence interval (CI) values based on the demographic and clinical variables for the development of the composite MACEs during the duration of the diseases. In these analyses, we categorized for potential confounders: sex, age (< 40 and ≥ 40 years), BMI (< 30 kg/m^2^/ ≥ 30 kg/m^2^), smoking and alcohol, diagnosis (others/r-axSpA), BASDAI and ASDAS-CRP (non-high/high risk).

IBM SPSS statistics (version 22.0, IBM Corp., Armonk, NY, USA) was used for statistical analysis. The significance level (*p* value) was determined at 0.05 in all statistical analyses.

## Results

There were 1457 patients with a mean age of 45.7 ± 10.9 years in the study. Most patients (70.1%) were 40 years of age or older. The male-to-female ratio was 2.1 in the study group. The socio-demographic characteristics are given in Table 1.

**Table 1:** Socio-demographic and clinical characteristics of the study groups

	Overall (*n* = 1457)	Group 1 (*n* = 44)	Group 2 (*n* = 1413)	*p*
Age (year)^a^	45 (18–83)	54 (41–83)	44 (18–83)	< 0.001
Age group^b^				
≥ 40 years	1021 (70.1)	44 (100.00)	977 (69.1)	< 0.001
< 40 years	436 (29.9)	0 (0)	436 (30.9)	
Sex^b^				
Female	477 (32.7)	14 (24.14)	463 (33.1)	0.198
Male	980 (67.3)	44 (75.86)	936 (66.9)	
BMI (kg/m^2^)^a^	26.8 (5.2–57.1)	28.5 (22.0–36.6)	26.8 (15.2–57.1)	0.106
BMI group^b^				
≥ 30 kg/m^2^	356 (24.4)	14 (31.8)	342 (24.2)	0.284
< 30 kg/m^2^	1101 (75.6)	30 (68.2)	1071 (75.8)	
Smoking^b^				
Current smoker	529 (36.7)	13 (29.5)	516 (36.9)	0.345
Alcohol^b^				
Current consumer	114 (8.3)	8 (20.5)	106 (7.9)	0.009
Marital status^b^				
Married	1204 (82.6)	38 (86.4)	1166 (82.5)	0.686
Single/widowed/divorced	253 (17.4)	6 (13.6)	247 (17.5)	
Educational status^b^				
Illiterate/primary	452 (31.0)	14 (31.8)	438 (31.0)	0.722
Secondary/high school	673 (46.2)	23 (52.3)	650 (46.0)	
College/university/doctorate	331 (22.7)	7 (15.9)	324 (22.9)	
Geographical regions^b^				
Marmara	373 (25.6)	6 (13.6)	367 (26.0)	0.098
Aegean	254 (17.4)	10 (22.7)	244 (17.3)	
Mediterranean	203 (13.9)	7 (15. 9)	196 (13.9)	
Central Anatolia	349 (24.0)	14 (31.8)	335 (23.7)	
Black Sea	140 (9.6)	7 (15.9)	133 (9.4)	
Eastern Anatolia	85 (5.8)	0 (0)	85 (6.0)	
South Eastern Anatolia	52 (3.6)	0 (0)	52 (3.7)	
Comorbidities^b^				
Hypertension	201 (13.8)	27 (61.4)	174 (12.7)	< 0.001
Diabetes mellitus	105 (7.2)	10 (22.7)	95 (6.9)	0.001
Chronic renal failure	24 (1.6)	5 (11.6)	19 (1.4)	0.001
Dyslipidemia	61 (4.2)	15 (48.4)	46 (6.9)	< 0.001
COPD	46 (3.2)	3 (6.8)	43 (3.1)	0.160
Malignancy	7 (0.5)	2 (4.5)	5 (0.4)	0.018
Valvular heart disease	10 (0.7)	1 (2.3)	9 (0.6)	0.265

Group 1 and 2: patients with and without major adverse cardiovascular event (cardiovascular disease)

*BMI* body mass index, *COPD* chronic obstructive pulmonary disease

^a^Median (min − max)

^b^*n* (%)

We found 44 patients (3%) with any MACE during the median disease duration of 126.8 months (Group 1). The distribution of these diseases was coronary artery disease (*n* = 42), congestive heart failure (*n* = 4), peripheral vascular disorders (*n* = 6), and cerebrovascular events (*n* = 4). The prevalence rates of CVD were 4.7%, 4.3%, 3.2%, and 0.6% in patients with PsA, peripheral SpA, R-axSpa, and Nr-axSpA, respectively. There were significant differences in the age of the patients, the age groups, sex, and alcohol status between the groups (*p* < 0.05) (Table 1). The patients in Group 1 were significantly older (*p* < 0.001). No patient was younger than 40 years in Group 1 (*p* < 0.001). The proportion of male patients was significantly higher in Group 1 than in Group 2 (0.014). There were more patients with current usage of alcohol in Group 1 than in Group 2 (*p* = 0.009). The other variables were similar in the groups (*p* > 0.05) (Table 1).

We detected significant differences in the frequencies of the comorbidities between the groups (*p* < 0.05) (Table 1). Hypertension (*n* = 201, 13.8%) and diabetes mellitus (*n* = 105, 7.2%) were the most frequent two diseases in the overall patients. In Group 1, there were significantly more patients with hypertension, diabetes mellitus, chronic renal failure, dyslipidemia, and malignancy than in Group 2 (*p* < 0.001, *p* = 0.001, *p* = 0.001, *p* < 0.001, and *p* = 0.018).

In the entire population, smoking (36.7%) and obesity (24.4%) were the most prevalent traditional CV risk factors, followed by hypertension (13.8%) (Table 1).

The disease-related characteristics of the study group are given in Table 2. The median duration of the diseases was significantly higher in Group 1 than in Group 2 (139 months vs. 126.8 months, *p* = 0.063). In the overall study group, r-axSpA was the most frequent phenotype of SpA seen in 1090 patients (74.8%), followed by nr-axSpA (11.6%). The distribution of the SpA phenotypes was similar in the groups (*p* = 0.328).

**Table 2:** Disease-related characteristics of the study groups

	Overall (*n* = 1457)	Group 1 (*n* = 44)	Group 2 (*n* = 1413)	*p*
Duration of disease (month)^a^	126.8 (0.3–763)	139 (40.6–763.0)	126.8 (0.3–577.3)	0.063
Diagnosis^b^				
R-axSpA	1090 (74.8)	35 (79.5)	1055 (74.7)	0.328
Nr-axSpA	169 (11.6)	1 (2.3)	168 (11.9)	
Peripheral SpA	46 (3.2)	2 (4.5)	44 (3.1)	
PsA	128 (8.8)	6 (13.6)	122 (8.6)	
Enteropathic SpA	19 (1.3)	0 (0)	19 (1.3)	
Unclassified	5 (0.3)	0 (0)	5 (0.4)	
HLA B-27^b^				
Positive	272 (18.7)	5 (11.4)	267 (18.9)	0.423
Negative	132 (9.1)	5 (11.4)	127 (9.0)	
Unknown	1053 (72.3)	34 (77.3)	1019 (72.1)	

Group 1 and 2: patients with and without major adverse cardiovascular event (cardiovascular disease)

*R-axSpA* radiographic axial spondyloarthritis, *Nr-axSpA* non-radiographic axial spondyloarthritis, *SpA* spondyloarthritis, *PsA* psoriatic arthritis

^a^Median (min − max)

^b^*n* (%)

We found no significant differences in the laboratory investigations except triglyceride and glomerular filtration rate (Table 3). The patients in Group 1 had significantly higher triglyceride values and lower glomerular filtration rate values than those in Group 2 (*p* = 0.010 and *p* = 0.010). The values of ESR and CRP were similar in the groups (*p* = 0.532 and *p* = 0.618).

**Table 3:** Laboratory investigations in the groups

	Group 1 (*n* = 44)	Group 2 (*n* = 1413)	*p*
ESR (mm/h)^a^	15 (2–53)	14 (1–103)	0.532
CRP (mg/dL)^a^	3.1 (0.8–57)	3.3 (0.1–168)	0.618
LDL (mg/dL)^a^	105 (50–242)	115 (40–400)	0.440
HDL (mg/dL)^a^	44 (26–70)	47 (22–153)	0.081
Cholesterol (mg/dL)^b^	202.7 ± 72.8	184.3 ± 55.8	0.439
Triglyceride (mg/dL)^b^	208.3 ± 133.7	144.7 ± 95.4	0.010
GFR (mL/min)^a^	95.2 (29.9–167.9)	108.9 (0.3–518)	0.010

Group 1 and 2: patients with and without major adverse cardiovascular events (cardiovascular disease)

*ESR* erythrocyte sedimentation rate, *CRP* C-reactive protein, *LDL* low-density lipoprotein, *HDL* high-density lipoprotein, *GFR* glomerular filtration rate

^a^Median (min − max)

^b^Mean ± standard deviation

The disease activity scores are summarized in Table 4. The comparison of the disease severity based on the BASDAI and ASDAS–CRP revealed no significant differences between the groups (*p* = 0.908 and *p* = 0.303). The scores for the other scales were similar in the groups, except for DAPSA (*p* = 0.042).

**Table 4:** Disease activity scores in the study groups

	Group 1 (*n* = 44)	Group 2 (*n* = 1413)	*p*
BASDAI^a^	3.4 (0–10)	3.2 (0–9.9)	0.675
BASDAI group^b^			
High disease activity	14 (31.8)	467 (33.1)	0.908
Normal	25 (56.8)	812 (57.5)	
Unknown	5 (11.4)	133 (9.4)	
ASDAS-CRP^a^	2.5 (0.4–4.2)	2.3 (0–5.8)	0.320
ASDAS-CRP group^b^			
High disease activity	29 (65.9)	768 (54.4)	0.303
Normal	13 (29.5)	577 (40.9)	
Unknown	2 (4.5)	67 (4.7)	
ASDAS-ESR^a^	2.56 (1.0–5.0)	2.4 (0.3–5.3)	0.231
BASMI^a^	4 (0–7)	2 (0–10)	0.008
MASES^a^	2.5 (05.5)-11)	2 (0–13)	0.621
BASFI^a^	3 (0.8–10)	2.9 (0–10)	0.402
VAS patient global^a^	50 (8–90)	50 (0–100)	0.602
VAS pyhsician global^a^	40 (5–90)	40 (0–100)	0.420
VAS pain^a^	50 (10–90)	40 (0–100)	0.096
VAS fatigue^a^	50 (0–90)	40 (0–100)	0.389
DAPSA^a^	6.2 (4.8–21)	16.8 (3–76)	0.042
PASI^a^	0 (0–0.4)	0 (0–72)	0.576
Symptom severity score^a^	3 (0–9)	3 (0–12)	0.600
Fibromyalgia severity score^a^	4 (0–26)	5 (0–29)	0.839
Tender joint score (0–44)^a^	4.5 (0–44)	2 (0–44)	0.282
Tender joint score (0–68)^a^	0 (0–4)	2 (0–44)	0.214
Swollen joint score (0–44)^a^	0 (0–4)	0 (0–21)	0.214
Swollen joint score (0–68)^a^	0 (0–4)	0 (0–21)	0.788

Group 1 and 2: patients with and without major adverse cardiovascular events (cardiovascular disease)

*BASDAI* Bath Ankylosing Spondylitis Disease Activity Index, *ASDAS-CRP* Ankylosing Spondylitis Disease Activity Score with C-reactive protein, *ASDAS-ESR* Ankylosing Spondylitis Disease Activity Score with erythrocyte sedimentation rate, *BASMI* Bath Ankylosing Spondylitis Metrology Index, *MASES* Maastricht Enthesopathy Score, *BASFI* Bath Ankylosing Spondylitis Functional Index, *VAS* Visual Analog Scales, *DAPSA* Disease Activity Index for Psoriatic Arthritis, *PASI* Psoriasis Area and Severity Score

^a^Median (min − max)

^b^*n* (%)

The medications used to treat the patients are given in Table 5. There was no difference in the patients with drug switches between the groups (*p* = 0.662).

**Table 5:** Medications used in the study groups

Medications^a^	Group 1 (*n* = 44)	Group 2 (*n* = 1413)	*p*
T lymphocyte inhibitors			
Abatacept	0 (0)	1 (0.1)	0.521
Tumor necrosis factor (TNF) inhibitors			
Adalimumab	18 (40.9)	595 (42.1)	0.529
Etanercept	15 (34.1)	485 (34.3)	0.527
Golimumab	14 (31.8)	354 (25.1)	0.283
Infliximab	12 (27.3)	253 (17.9)	0.131
Certolizumab pegol	3 (6.8)	138 (9.8)	0.442
Janus kinase inhibitors			
Tofacitinib	0 (0)	2 (0.1)	0.514
IL-17 inhibitors			
Ustekinumab	0 (0)	15 (1.1)	0.423
Secukinumab	7 (15.9)	141 (10.0)	0.216
Hydroxychloroquinine	1 (2.3)	22 (1.6)	0.489
Pyrimidine synthesis inhibitors			
Leflunomide	2 (4.5)	32 (2.3)	0.317
Sulfasalazine	19 (43.2)	824 (58.3)	0.098
Methotrexate	8 (18.2)	199 (14.1)	0.373
Steroids	4 (9.1)	124 (8.8)	0.554
Nonsteroid anti-inflammatory drugs	32 (72.7)	1132 (80.1)	0.349
Main medication groups			
IL-17 inhibitors	7 (16.7)	154 (11.1)	0.501
TNF inhibitors	40 (95.2)	1298 (93.8)	1.0
Switching			
No switch	5 (11.4)	110 (7.8)	0.662
1 switch	11 (25.0)	342 (24.2)	
2 or more switches	28 (63.6)	961 (68.0)	

Group 1 and 2: patients with and without major adverse cardiovascular event (cardiovascular disease)

^a^*n* (%)

The univariate Cox proportional regression analysis revealed that age, hypertension, diabetes mellitus, dyslipidemia, and cancer history were the significant risk factors for the development of CVD. Nevertheless, the multivariate analysis showed that hypertension (HR = 3.147, 95% CI 1.461–6.778, *p* = 0.003), dyslipidemia (HR = 3.476, 95% CI 1.631–7.406, *p* = 0.001), and cancer history (HR = 5.852, 95% CI 1.189–28.810, *p* = 0.030) were the independent predictors for CVD in the study group (Table 6).

**Table 6:** Univariate and multivariate Cox proportional regression analysis for major adverse cardiovascular events during the duration of the diseases

Parameter	Reference	Risk factor	Univariate	Multivariate	
			*p*	HR (95% CI)	*p*
Age			0.007	1.001 (0.956–1.049)	0.956
Sex	Female	Male	0.410	0.628 (0.226–1.746)	0.373
BMI group	< 30 kg/m^2^	≥ 30 kg/m^2^	0.958	0.731 (0.315–1.699)	0.327
Smoking	Non-/ex-smoker	Smoker	0.128	0.598 (0.223–1.601)	0.306
Alcohol	Non-/ex-consumer	Consumer	0.158	0.703 (0.231–2.142)	0.535
Hypertension	Absent	Present	< 0.001	4.994 (1.966–12.683)	0.001
Diabetes mellitus	Absent	Present	0.297	0.629 (0.206–1.920)	0.416
Chronic renal failure	Absent	Present	0.013	1.241 (0.182–8.445)	0.826
Dyslipidemia	Absent	Present	< 0.001	2.960 (1.155–6.776)	0.024
Chronic obstructive pulmonary disease	Absent	Present	0.330	0.809 (0.131–4.982)	0.819
Malignancy	Absent	Present	0.003	2.293 (0.131–40.212)	0.570
Phenotypes of SpA	Diagnoses other than r-axSpA	r-axSpA	0.365	0.788 (0.309–1.930)	0.581
BASDAI group	Non-high risk	High-risk	0.404	0.750 (0.325–1.727)	0.498
ASDAS-CRP group	Non-high risk	High-risk	0.063	2.158 (0.834–5.587)	0.113

*HR* hazard ratio, *CI* confidence interval, *BMI* body mass index, *R-axSpA* radiographic axial spondyloarthritis, *SpA* spondyloarthritis, *BASDAI* Bath Ankylosing Spondylitis Disease Activity Index, *ASDAS-CRP* Ankylosing Spondylitis Disease Activity Score with C-reactive protein

## Discussion

This nationwide registry-based study using the BioSTAR database showed that the CVD prevalence rate in SpA patients was 3% within the median disease duration of 126.8 months. Notably, hypertension, dyslipidemia, and a history of cancer emerged as significant risk factors for CVD in this cohort. This research represents one of the pioneering studies analyzing CVD prevalence across all SpA phenotypes within the Turkish population.

The prevalence rates for CVD in patients with inflammatory joint diseases show considerable variances across studies. Yagensky et al. [34] reported prevalence rates of 8.7% and 12.8% for SpA and PsA. Similar rates were reported by the other studies [32, 33, 35, 36]. Interestingly, the prevalence rates in López-Medina’s and Kao's studies were relatively lower than the previously documented rates [1, 6]. In the ASAS–COMOSPA study, the patients from the Mediterranean area had lower CVD rates (1.8% and 1.3% for ischemic heart disease and stroke) than those in Northern European countries (6.2% and 2.5% for ischemic heart disease and stroke), indicating potential regional disparities [1]. Kao et al. [6] highlighted a 2.9% CVD prevalence in newly diagnosed AS patients, suggesting a potential link between increased prevalence and older age or prolonged disease duration [6]. In the current study, the overall prevalence rate for CVD in patients with SpA was 3.0%. The variations in the distribution of SpA phenotypes, geographical areas, and disease activity might be the reasons for such differences. Although older people are more prone to CVD, the possible association of increased disease duration or severity with a higher prevalence of CVD in patients with SpA warrants clarification through prospective studies.

The association between traditional CV risk factors and SpA has been investigated for decades [5, 6]. It is generally believed that SpA patients present with an atherogenic metabolic profile, with chronic systemic inflammation potentially exacerbating both past and future CV events. Notably, indicators of systemic inflammation such as uveitis or elevated CRP levels have been linked to increased CVD in ankylosing spondylitis patients [8, 37, 38]. In that way, these patients might see atherosclerotic events more frequently [6]. However, conflicting outcomes in some studies [39–41] underscore methodological differences and patient characteristics, contributing to varied results.

Previous studies have had different prevalence rates of the traditional CV risk factors. In our study, smoking and obesity emerged as prominent risk factors, while other CV risk factors were less prevalent. Similar rates have been reported in the Moroccan population, albeit in a smaller SpA patient cohort [3]. Nevertheless, hypercholesterolemia was the most frequent risk factor seen in more than half of SpA patients [34]. Hypertension was the most common risk factor in almost half of the Norwegian patients with inflammatory joint diseases [35]. In varying prevalence rates from 19.2% to 33.8%, hypertension was seen in patients with different SpA phenotypes in the ASAS–COMOSPA study [1]. Although the prevalence rates of each risk factor have shown considerable variations in each study, socio-demographic, environmental, behavioral, and clinical characteristics might be used to explain such differences [1, 5]. Consequently, assessing data separately within each country or population becomes crucial when evaluating CV risk in SpA patients.

Several authors investigated the reasons for mortality in inflammatory joint diseases, including rheumatoid arthritis, PsA, and axSpA. The authors reported cardiovascular and respiratory diseases as the two leading causes of death in patients with axSpA in the Norwegian Cardio-Rheuma Register [9, 32]. Nevertheless, CARdiovascular in rheuMAtology (CARMA) prospective study showed that patients with SpA were more likely to have an increased risk for CVE. In contrast, the corresponding CV mortality was lower than the estimated rates [32, 33]. The length of follow-up is also a vital factor for detecting more accurate data about mortality. As the duration of follow-up increases, it is expected to see more deaths in aging patients with variable severity of chronic inflammatory joint diseases.

Disease activity has been proposed as a potential risk factor for major adverse CV events (MACEs) in SpA patients, with higher disease activity potentially linked to an increased number of traditional CV risk factors [2, 6]. While our study did not specifically analyze the correlation between the number of CV risk factors and disease characteristics, no significant association was found between disease activity based on BASDAI and ASDAS–CRP groups and CVD development.

The association between drugs and MACEs in SpA patients is another speculative issue [6, 42]. Despite the cardiac toxicities, the antiinflammatory action mechanisms of these medications might attenuate the underlying atherogenic potential and serve the cardioprotection via reducing the disease activity and controlling systemic inflammation for SpA patients with different phenotypes [5, 43, 44]. Nevertheless, the association between the risk of CV mortality and nonsteroidal antiinflammatory drug usage in SpA patients remains controversial [45]. The highly selective COX-2 inhibitors and steroids were the other speculative drugs leading to a higher CVD risk [44]. Kao et al. [6] reported the negative impact of selective COX-2 inhibitors and corticosteroids on developing MACEs in AS patients. However, the results conflicted because of methodological insufficiencies [6]. TNF inhibitors were associated with a reduced risk of CVD according to the retrospective follow-up analysis of 5046 SpA patients [5]. Secukinumab and ixekizumab were the selective IL-17A inhibitors that improved the degree of systemic inflammation without negatively impacting traditional CV risk factors [5, 46, 47]. There were questionable relationships between the targeted synthetic DMARDs and MACE risk in SpA patients [45, 48–50]. Based on the systematic reviews and meta-analyses, the evidence that specific types of medications were associated with a higher risk of CVD is still being determined due to methodological problems [41, 44]. We did not analyze the impact of the patient's past and current medication history on the development of CVD, considering the difficulty in collecting data about the quantity of the drugs [2]. The uncertainty regarding the possible interconnections between the disease activity, comorbidities, and the indications for the drugs might influence the results of the previous studies [9, 47]. Although medication adherence was obtained using the patients' medical files or the patient-reported outcomes retrospectively, prospective large-scale studies might be more beneficial for robust conclusions.

Nationwide data about SpA patients with different phenotypes was the main strength of this study. Although the inclusion of all different phenotypes might be regarded as the study's heterogeneity, variability in SpA phenotypes and disease characteristics within the studied population could influence the applicability of our findings to diverse SpA subgroups to understand the overall risk of CVD in this patient group. We also aimed to compare the differences in the prevalence rates of each phenotype. The insufficient number of patients in some phenotypes prevented such comparisons. Besides, due to the study's cross-sectional design, we could not discriminate whether CVD was present before or after the diagnosis of SpA.

The cross-sectional nature of our study restricted our ability to establish the temporal sequence between the onset of CVD and the diagnosis of SpA, which is the study's major limitation. In that way, CVD occurrences predated or followed the diagnosis of SpA, the complex interplay between disease-specific factors, limitations inherent in the dataset, and the potential influence of unmeasured confounders or variables not included or analyzed in the study.

Another limitation might be the potential underestimation of CVD prevalence due to the likelihood of subclinical cases and unregistered hospital admissions.

In conclusion, this nationwide, cross-sectional registry study revealed that CVD risk in patients with SpA was relatively low compared to the previous rates. Among the traditional CV risk factors, hypertension and dyslipidemia were the independent predictive factors for the development of CVD. Besides, cancer history was another independent risk factor for CVD. Large-scale, prospective studies are needed to clarify the controversial issues in predicting CVD risk.
